# Properties of Resveratrol: *In Vitro* and *In Vivo* Studies about Metabolism, Bioavailability, and Biological Effects in Animal Models and Humans

**DOI:** 10.1155/2015/837042

**Published:** 2015-06-28

**Authors:** J. Gambini, M. Inglés, G. Olaso, R. Lopez-Grueso, V. Bonet-Costa, L. Gimeno-Mallench, C. Mas-Bargues, K. M. Abdelaziz, M. C. Gomez-Cabrera, J. Vina, C. Borras

**Affiliations:** ^1^Department of Physiology, Faculty of Medicine, University of Valencia, INCLIVA, Blasco Ibáñez Avenue 15, 46010 Valencia, Spain; ^2^Department of Physiotherapy, Faculty of Physiotherapy, University of Valencia, Gascó Oliag Street 5, 46010 Valencia, Spain; ^3^Sports Research Centre, Miguel Hernández University of Elche, University Avenue, s/n, 03202 Elche, Alicante, Spain

## Abstract

Plants containing resveratrol have been used effectively in traditional medicine for over 2000 years. It can be found in some plants, fruits, and derivatives, such as red wine. Therefore, it can be administered by either consuming these natural products or intaking nutraceutical pills. Resveratrol exhibits a wide range of beneficial properties, and this may be due to its molecular structure, which endow resveratrol with the ability to bind to many biomolecules. Among these properties its activity as an anticancer agent, a platelet antiaggregation agent, and an antioxidant, as well as its antiaging, antifrailty, anti-inflammatory, antiallergenic, and so forth activities, is worth highlighting. These beneficial biological properties have been extensively studied in humans and animal models, both *in vitro* and *in vivo*. The issue of bioavailability of resveratrol is of paramount importance and is determined by its rapid elimination and the fact that its absorption is highly effective, but the first hepatic step leaves little free resveratrol. Clarifying aspects like stability and pharmacokinetics of resveratrol metabolites would be fundamental to understand and apply the therapeutic properties of resveratrol.

## 1. Background

**Figure 1:**
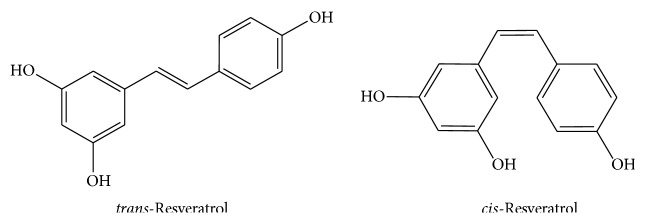
Chemical structures of* trans*-resveratrol and* cis*-resveratrol.

Resveratrol (3,5,4′-Trihydroxystilbene) is a natural polyphenol with a stilbene structure. Its chemical structure was characterized in 1940 by Takaoka, who isolated it from the root of* Veratrum grandiflorum* [1]. However, it has been present in medicinal preparations, such as* darakchasava *or* manakka* [2], for more than 2000 years. Its basic structure consists of two phenolic rings bonded together by a double styrene bond, which forms the 3,5,4′-Trihydroxystilbene (molecular weight 228.25 g/mol). This double bond is responsible for the isometric* cis*- and* trans*-forms of resveratrol (Figure 1). It is worth mentioning that the* trans*-isomer is the most stable from the steric point of view [3].

There are many synthetic and natural analogues of resveratrol, as well as adducts, derivatives, and conjugates, including glucosides [4].

The synthesis of resveratrol decreases regularly during the grape ripening process, which explains the increasing susceptibility of mature fruits to infection by* Botrytis cinerea* [5].

Resveratrol is a phytoalexin. These chemicals are characterized by their low molecular weight and their ability to inhibit the progress of certain infections. The accumulation of these substances in plants is produced by a mechanism of resistance to parasites and other adverse conditions, like fungal infection, UV radiation, chemical substances and, in general, stressful factors for the plant [5–7]. In fact, resveratrol is produced by more than 70 species of plants in response to such stressful situations [8].

The concentration of resveratrol in plants depends on various factors. For example, in vines, the two most important factors are the weather and presence of fungus [9–12].

Resveratrol can be found in some fruits, which are part of the human diet, such as blueberries (*Vaccinium spp*.), blackberries (*Morus spp*.), and peanuts (*Arachis hypogaea*) [13, 14]. However, red wine is the main source of resveratrol in the Mediterranean diet.

Resveratrol content in red wine comes from grapes (*Vitaceae*). In particular, the richest sources are the skin, seeds, petioles, and woody parts [15]. For that reason, red wine is richer in resveratrol than white wine, because during the production of red wine, parts of the grape where resveratrol is concentrated are macerated. This does not happen in white wine [16, 17]. Alcohol formation during grape fermentation facilitates its solubility and thus its extraction.

*Cis*- and* trans*-isomers coexist in plants and in wine. However,* cis*-resveratrol has never been found in grape extract [18, 19]. The* trans*-isomer appears to be the more predominant and stable natural form.* Cis*-isomerisation can occur when the* trans*-isoform is exposed to solar [20] or artificial light or ultraviolet radiation [21] at a wavelength of 254 [22] or 366 nm [23].

Although less important in our culture, the richest source of resveratrol is the* Polygonum cuspidatum* herb, whose root extract has played a very important role in Japanese and Chinese traditional medicine. In fact, it is the main active ingredient in* ko-jo-kon*, which is used in the treatment of several cardiovascular diseases [24].

*Veratrum grandiflorum* has a high content of resveratrol in leaves, when the plant is damaged by any chemical treatment [25]. Furthermore, the roots and rhizomes of* Veratrum formosanum* are also rich in resveratrol and, in fact, a preparation of this plant has been traditionally used in east Asia to treat hypertension [26].

## 2. Absorption, Metabolism, and Bioavailability

### 2.1. Absorption

The chemical structure of resveratrol leads to low water solubility (<0.05 mg/mL), which affects its absorption. In order to increase its solubility, ethanol (50 mg/mL) or organic solvents may be used.

It is important to highlight the ability of resveratrol to form a wide range of organic molecular complexes. Sterification of hydroxyl groups with aliphatic molecules can also be employed as a tool to increase its intestinal absorption and cellular permeability. For example, resveratrol acetylation can increase its absorption and its cellular capture without loss of activity [27–29].

At the intestinal level, resveratrol is absorbed by passive diffusion or forming complexes with membrane transporters, such as integrins. Once in the bloodstream, resveratrol can be found essentially in three different forms: glucuronide, sulfate, or free. The free form can be bound to albumin and lipoproteins such as LDL (low-density lipoprotein). These complexes, in turn, can be dissociated at cellular membranes that have receptors for albumin and LDL, leaving the resveratrol free and allowing it to enter cells. Resveratrol's affinity for albumin suggests that it could be a natural polyphenolic reservoir, playing an important role in its distribution and bioavailability [30].

Due to its chemical characteristics, resveratrol can interact with fatty acids. Recent studies* in vitro* show that more than 90% of free* trans*-resveratrol binds to human plasma lipoproteins. This binding is also found* in vivo*, as shown by the presence of dietary polyphenolic compounds detected in isolated LDL in blood samples of healthy human volunteers [31, 32].

Fatty acids facilitate a lipophilic environment, which favors resveratrol binding [33]. Normally they are employed as vectors because of their high affinity for the liver and their efficient cellular uptake, resulting from specific interactions with transmembrane transporters.

### 2.2. Metabolism

Phase II metabolism of resveratrol or its metabolites occurs in liver. There is enterohepatic transport in bile, which may result in some returning cycles to the small intestine [34]. Furthermore, resveratrol is able to induce its own metabolism, increasing the activity of phase II hepatic detoxifying enzymes [35].

**Figure 2:**
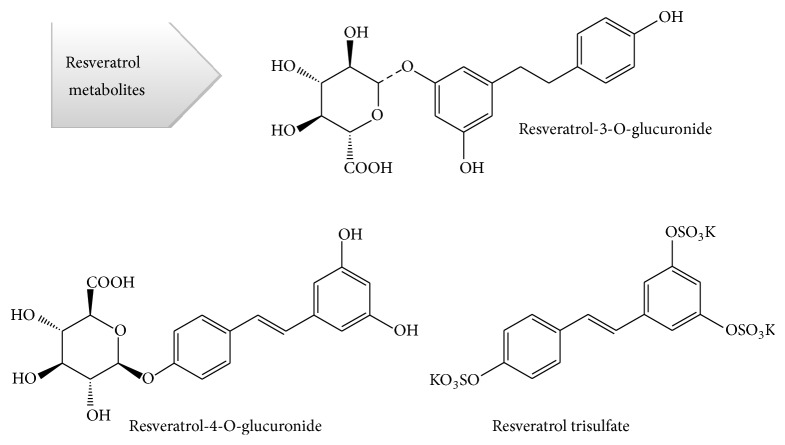
Resveratrol metabolites.

Resveratrol has a high metabolism, leading to the production of conjugated sulfates, glucuronides (Figure 2), which retain some biological activity [36], and up to five different metabolites present in the urine: resveratrol monosulfate, two isomeric forms of resveratrol monoglucuronide, monosulfate dihydroresveratrol, and monoglucuronide dihydroresveratrol. However, the nature and quantity of these metabolites can differ between subjects due to interindividual variability [37–39].

*Cis*-metabolites have been identified in human urine samples, mainly as* cis*-resveratrol-4′-sulfate,* cis*-resveratrol-3-O-glucuronide, and* cis*-resveratrol-4′-O-glucuronide [32, 40]. Most research has been performed with* trans*-isomer due to the instability of* cis*-isomer [20]. However, data indicate that both of them can have different biological effects [41–43].

Other dietary flavonoids, such as quercetin, may inhibit resveratrol sulphation and glucuronidation in the liver and duodenal tissue, increasing its bioavailability [44].

### 2.3. Bioavailability

Resveratrol exhibits lipophilic characteristics, which lead to a high absorption. However, it should be noted that resveratrol absorption can vary depending on the way it is consumed and the kind of food ingested [45].

Low bioavailability of resveratrol is a factor that may reduce the efficacy of resveratrol. Although* in vitro* studies show a high efficacy in biologically beneficial effects of resveratrol in cells, it is known that its distribution in tissues is very low. Consequently,* in vitro* studies must be interpreted with caution when trying to extrapolate its effect in* in vivo* studies.

Despite its low bioavailability, resveratrol shows efficacy* in vivo*. This may be explained by the conversion of both sulfates and glucuronides again to resveratrol in target organs such as the liver [44, 46]. Another possible explanation could be the enterohepatic recirculation of resveratrol metabolites, followed by its deconjugation in the small intestine and its reabsorption [47]. Finally,* in vivo* effects could be explained by the activity of its metabolites.

Glucuronidation of the* cis*-form is faster (5–10 times) than that of the* trans*-form, thus leading to a lower bioavailability of the* cis*-form [44].

The presence of hydroxyl groups allows polyphenols to associate with proteins and other nutrients. The solubility of these compounds determines its physiological effects. Thus, complexes including these macronutrients and polyphenols, which maintain solubility, can be absorbed in the small intestine, while insoluble complexes are eliminated in feces, reducing their availability [48].

Two of the first human studies on the absorption and bioavailability of resveratrol used a single oral dose treatment of 25 mg [37, 49]. Despite the use of high sensitivity methods and a specific molecular analysis, it was difficult to detect the nonmetabolized resveratrol in circulating plasma. Approximate calculations showed maximal concentrations of <10 ng/mL (≈40 nM), 0.5–2 hours after the oral dose. Estimates of the plasmatic concentrations of resveratrol plus total metabolites were considerably higher, around 400–500 ng/mL (≈2 *μ*M), indicating a very low oral bioavailability of free resveratrol, but a significant one of its metabolites [37, 49].

Urinary excretion of total metabolites after a radio-labeled dose was administered showed that about 75% of orally or intravenously administered resveratrol was absorbed [37]. This is an unusually high absorption for a dietary polyphenol, particularly in view of the poor aqueous solubility of this compound.

Several approaches have been used to increase the bioavailability of resveratrol in humans. The dose concentration curve seems to be a logical method, and it has been examined in two studies, with a dose range from 25 to 1000 mg [39, 50], covering the wide range used in chemoprevention studies. The absorption in these cases reached a maximum of between 0.3 and 2.4 *μ*M, which does not reach the anticancer properties found at concentrations higher than 5 *μ*M. Furthermore, in these studies an increase in the bioavailability of resveratrol during the treatment was found and a lack of metabolism saturation with the highest concentrations (500 mg/mL) [39]. Nevertheless, other studies in rats, which were administered resveratrol for 15–20 weeks, showed that a saturation of metabolites exists, and it leads to an increase of resveratrol in plasma and thus in tissues [51–53].

A pharmacokinetic study of repeated doses over two days concludes that tolerance is good, concentrations in plasma do not increase over time and even decrease, and the bioavailability is higher when administered in the morning [50].

Vitaglione et al. carried out an interesting study on the bioavailability of free* trans*-resveratrol present in red wine in humans [45]. Subjects were randomly divided into three experimental groups, consuming different types of food and red wine. The first group consumed 300 mL of red wine with a free* trans*-resveratrol content of 0.82 mg/L with a meal consisting of beef, egg, bread, corn oil, and French fries. The second group consumed 600 mL of red wine containing 3.2 mg/L free* trans*-resveratrol while fasting (before breakfast). Lastly, the third group consumed two different meals with different lipid content and 600 mL of red wine, which assured a free* trans*-resveratrol total ingestion of 0.48 mg. The authors concluded that the kind of food does not affect resveratrol bioavailability and found much variability between individuals. However, these results are inconsistent with those of other studies, in which a high-fat meal decreased its absorption [54]. Thus, we conclude that the different sample processing methods and the kind of analysis are the key to detect both free or conjugated resveratrol [45].

## 3. Biological Properties

The beneficial properties of the phenolic compounds present in grapes and wine have been studied after the discovery of the “French Paradox.” This term refers to the fact that in north France there is a high intake of saturated fat but low mortality from coronary heart disease compared to other countries where the same high saturated fat intake exists, being the Paradox attributable to high wine consumption [55]. In fact, there are more and more studies dealing with the ability of grape polyphenols and red wine to protect against different types of diseases [56, 57]. Resveratrol is one of the most studied red wine molecules and, in fact, there are more than 1000 references about its properties in the bibliography. Some of these studies* in vivo* and* in vitro* are described in Table 1.

Because of its chemical and physical features, resveratrol can either cross passively cell membranes or interact with membrane receptors. Therefore, it may interact with extracellular and intracellular molecules. For this reason, its mechanism of action at the cellular level may be triggered by either activating signaling pathways when binding to cell membrane receptors, activating intracellular mechanisms, or even developing its effects inside the nucleus.

### 3.1. Phytoestrogenic Properties

In fact, resveratrol is able to bind to estrogen receptors* alpha* and* beta* (ER-*α* and ER-*β*) with similar affinities, but this interaction is 7000 times less powerful than that of estradiol [58]. Molecular studies have shown that the union of resveratrol to ER-*α* is stereoselective, that is, that the* trans*-isomer shows more affinity for this receptor than the* cis*-isomer [59].

**Figure 3:**
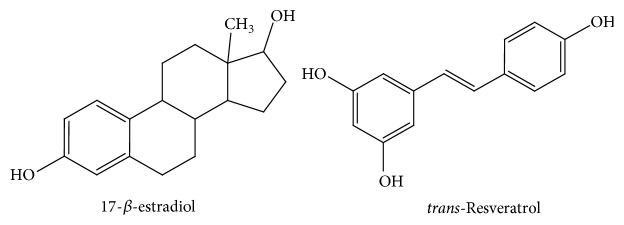
Comparison of the chemical structures of* trans*-resveratrol and 17-*β*-estradiol.

The chemical structure of resveratrol is similar to that of 17-*β*-estradiol (Figure 3) or synthetic estrogens like diethylstilbestrol. Thus, several studies have been carried out in order to test its ability to act as a phytoestrogen [60–63].

Estrogens and phytoestrogens exert almost all of their effects through binding to estrogen receptors. When estrogen binds to its receptor, it activates the transcription of target genes. We found that antioxidant genes were upregulated by this mechanism [64, 65]. Resveratrol can bind to estrogen receptors and activate the transcription of such genes with similar concentrations to those required for its other biological effects. In this regard, Gehm et al. demonstrated in 1997 that resveratrol behaves as an estradiol analog [63]. They used MCF-7 cells, which are rich in estrogenic receptors. Maximal efficiency binding was at 10 *μ*M. This subsequently activated genes with estrogen responsive elements (ERE). Furthermore, to confirm that resveratrol starts ERE activation, they used estrogenic antagonists and the effect was inhibited. By contrast, it has also been shown that resveratrol, in its capacity of an estrogen receptor modulator, can also antagonize the effect of estradiol on increasing proliferation of MCF-7 cells, at higher doses [62].

Regarding its estrogenic activity, it has been shown that it does not have any effect on the growth and differentiation in the uterus of growing rats. In the same article, the authors did not find any effect of resveratrol on either radial bone growth, serum cholesterol levels, or animal body weight. This study concludes that resveratrol does not act as an agonist in rats at doses from 1 to 100 *μ*g/day. Even with higher doses (1000 *μ*g/day) the effect is insignificant and could also act as an estrogen antagonist [62].

### 3.2. Antioxidant Properties

Oxidative damage is involved in the pathogenesis of many important diseases, such as diabetes [66], cardiovascular diseases [67], neurodegenerative diseases [68], and cancer [69]. It also plays an important role in the aging process [70, 71]. Therefore, a great deal of attention has been focused on finding natural antioxidants, which could help in the treatment of all these diseases and, consequently, potential antioxidant effects of resveratrol have been studied in depth.

Its antioxidant activity has been determined in isolated rat brain mitochondria, which shows an inhibition of the mitochondrial respiration state when they are incubated with resveratrol. Furthermore, it inhibits the activity of complex III by competing with coenzyme Q. This fact is interesting because it determines its antioxidant activity in mitochondria, not only its activity in uptake capacity of unpaired electrons, but also by inhibiting a complex that generates free radicals [72].

Most published* in vitro* studies report using concentrations of resveratrol too high to be reached in the organism after red wine consumption. Therefore, it is very important to make sure that low plasma concentrations of free resveratrol are sufficient enough to be active as an antioxidant. In this regard, it has been shown that nutritionally relevant concentrations of resveratrol are able to decrease H_2_O_2_ levels in MCF-7 cells by inducing the expression of antioxidant genes, such as catalase [12] and manganese superoxide dismutase (MnSOD), through a mechanism that involves phosphatase and tensin homolog (PTEN) and proteinkinase-B (PKB or Akt) signaling pathway [73].

In the cardiovascular system it has been reported how this polyphenol, at a concentration of 20 *μ*M, can reduce the malondialdehyde content in blood mononuclear cells isolated* ex vivo* from healthy individuals [74]. Thus, resveratrol preincubation of bovine aortic smooth muscle cells was able to attenuate oxidized low-density lipoprotein- (oxLDL-) induced increases in reactive oxygen species (ROS) and H_2_O_2_ levels [75]. In another study performed in human blood platelets treated with peroxynitrite, resveratrol inhibited protein carbonylation and nitration, as well as lipid peroxidation [76].

Regarding other physiological systems and tissues, resveratrol has also been shown to protect primary hepatocytes in culture against oxidative stress damage by increasing the activities of catalase, superoxide dismutase, glutathione peroxidase, NADPH quinone oxidoreductase, and glutathione-S-transferase. Furthermore, it increases the level of nuclear factor (erythroid-derived 2)-like 2 (Nrf2) and induces its translocation to the nucleus [77]. This factor can activate genes with antioxidant responsive elements (ARE).

In rat spinal cord, resveratrol was shown to protect it from secondary spinal cord injuries via improving the energy metabolism system and inhibiting the lipid peroxidation, at a dose between 50 and 100 mg/kg, reaching the maximal effect after 48 h of the spinal cord injury [78]. In a related article, resveratrol protected rabbit spinal cord from ischemia-reperfusion injury by decreasing lipid peroxidation (at a dose of 10 mg/kg) and increasing nitric oxide (NO) release (at doses of 1 mg/kg and 10 mg/kg) [76, 79].

Regarding the musculoskeletal system, it has been described how, in young and old rats submitted to a 14-day muscle disuse by hindlimb suspension, resveratrol (at a dose of 12.5 mg/Kg) was able to diminish oxidative stress by increasing gastrocnemius catalase activity, MnSOD activity, and MnSOD protein content. Interestingly, resveratrol was also able to regain the muscle isometric force, but apoptotic markers were not modified [80]. Another similar article also deals with the ability of resveratrol to protect against muscle and bone alterations after disuse and suggests resveratrol as a physical exercise mimetic [81].

The ability of resveratrol to act as an antioxidant has also been found in a model of senescence-accelerated mice, where resveratrol at different dosages (25, 50, and 100 mg/Kg/day) for 8 weeks increased the activity of superoxide dismutase (SOD) and glutathione peroxidase (GPx), as well as diminishing malondialdehyde levels [82, 83].

Despite this antioxidant function, however, it can also suffer an autooxidation process, leading to the production of O_2_^∙−^, H_2_O_2_, and a complex mixture of semiquinones and quinones, which can become cytotoxic [84, 85]. The oxidized resveratrol molecule can generate complexes with copper that can fragment DNA [86].

### 3.3. Antitumor Effects

Resveratrol can interact with the *α*V*β*3 integrin receptor in MCF-7 cells (a breast-cancer cell line) inducing apoptosis [87]. Besides, it shows antagonist actions when binding to the aryl hydrocarbon receptor, which has immunosuppressive and carcinogenic activity in cells [88].

Nevertheless, these studies conclude that resveratrol inhibits cellular proliferation at concentrations within 10–30 *μ*M. In particular, the effect is locked in phase G/S2 of the cellular cycle, suggesting an inhibition in the enzymatic activities responsible for DNA duplication. These effects have been observed in a cell line of prostate cancer with a concentration of 25 *μ*M but not with 2.5 *μ*M [89].

Some studies show that it can exert its antitumor effects on the initiation, promotion, and progression of cancer in tumor cells [89]. In this regard, it has been shown how resveratrol at 15 *μ*M is able to inhibit cyclooxigenase 1 (COX-1), a very active enzyme involved in tumor progression. In addition, at 11 *μ*M, it induces phenotypic nonproliferative markers, like the reduction of the nitroblue tetrazolium activity. In the initiation of tumor cells, it acts to inhibit the formation of free radicals at 27 *μ*M on leukemia cells (HL-60). In hepatoma cells (Hepa LcLc7), it inhibits hepatic reductase activity, an enzyme which produces hepatic toxicity, at concentrations of 21 *μ*M. In addition, at 18 *μ*M, the incorporation of thymidine is inhibited, indicating the end of differentiation and thus the transformation to a nonproliferative phenotype [90]. In MCF-7 cells, 10 *μ*M resveratrol blocks the aryl hydrocarbon receptor obtaining beneficial effects against cancer, as it is reported that the activation of this receptor may be involved in some types of tumors [91]. The anticancer effect of resveratrol in MCF-7 has also been associated with BCL-2 and NF-kappa*β* [92]. Between 10 and 40 *μ*M, it induces apoptosis via p53 activation in human lymphoblast cell lines [93]. It can also inhibit ribonuclease reductase [94] or COX-2 activity [95]. For that reason, resveratrol has antitumor effects when administered* in vitro*.

*In vivo* studies show beneficial effects [96, 97]. For example, its preventive effect on the initiation of cancer has been determined in a skin cancer animal model, with a concentration between 1 and 25 *μ*M of resveratrol, and administrated twice a week [90]. Thus,* in vivo* studies support the antitumor beneficial effects previously seen in* in vitro* studies.

### 3.4. Cardiovascular Effects

Platelet aggregation is inhibited by resveratrol both* in vitro* and* in vivo*. There are studies that suggest that resveratrol, at concentrations of 0.1, 1, and 10 *μ*M binds to calcium channels producing 20, 30, and 50% inhibition of thrombin, respectively [98]. This is very beneficial for the cardiovascular system, due to its interference in the formation of blood clots.

Those effects on platelet aggregation showed in* in vitro* studies mentioned above have also been shown in an* in vivo* study in rabbits, when a dose of 4 mg/kg/day of resveratrol was administered [99].

Other cardiovascular effects attributed to resveratrol are the regulation of the accumulation of triglycerides and the regulation of the lipolysis in murine adipocytes. When human adipocyte cells (3T3-L1 and SGBS) are incubated with resveratrol at 100 *μ*M, a decrease in triglycerides is observed by an induction of lipolysis, activating adipose triglyceride lipase, and by inducing lipid mobilization [100]. Thus, authors suggest a possible treatment for obesity.

### 3.5. Other Biological Effects

In a cellular model of leucocytes (RBL-2H3 cells), it has been observed that resveratrol at 15 *μ*M has an antiallergenic effect by decreasing the *β*-hexosaminidase activity [101].

In human mesenchymal stem cells, resveratrol promotes a spontaneous osteogenesis, activating genes such as osteocalcin and RUNX2. It also prevents adipogenesis by repressing the expression of some genes such as* PPARγ2* and leptin [102], suggesting beneficial effects of resveratrol on bone regeneration.

Resveratrol has been shown to have beneficial effects on experimental diabetes. It improves the health status of diabetic rats induced with streptozotocin, by enhancing the energy metabolism and reducing protein breakdown [103].

## 4. Resveratrol, Sirtuins, and Aging

### 4.1. Invertebrates

It has been reported that resveratrol can extend lifespan in some organisms, such as the budding yeast* Saccharomyces cerevisiae*, involving a similar mechanism to that of calorie restriction (CR) [104]. Further studies have confirmed these results in the* Caenorhabditis elegans* nematode and the* Drosophila melanogaster* fly [105]. This effect has been shown to be mediated by the activation of sirtuin 2 [105], an enzyme induced, for example, by calorie restriction, physical exercise, and ethanol consumption [106]. Sirtuins (Sirt or Sir, from “silent information regulator”) belong to a family of enzymes with deacetylase activity [107]. These enzymes have the ability to modify covalently the histones that cover the DNA by deacetylation, inhibiting the transcription of certain genes. They can also activate or inhibit important enzymes by deacetylation. These enzymes can be activated by resveratrol consumption. Further studies seem to verify this capacity of resveratrol to increase lifespan using* Drosophila spp*. [108] and* C. elegans* [109]. In this context, a CR-sirtuin-prolonging lifespan association was established, in which a low-calorie diet would activate sirtuins and regulate mechanisms which extend lifespan. It was suggested that resveratrol could activate the same mechanisms as CR so that the new association was resveratrol-sirtuin-prolonging lifespan [104, 110]. However, in the past years, the role of Sir2 and resveratrol in aging and the relationship with CR have brought about controversy [96, 111, 112]. In fact, some authors postulate that resveratrol cannot activate Sirt2* in vivo* [111, 113] and that CR could increase fly lifespan regardless of Sirt2 activation [114]. Thus, a recent study performed on 30 and 130 *μ*M resveratrol pretreated honey bees showed that resveratrol was able to extend lifespan under normal conditions, but not under hyperoxia [82].

### 4.2. Vertebrates

In a recent study, which was carried out on five-year-old male grey mouse lemurs (*Microcebus murinus*), resveratrol decreased glycemia after an oral glucose loading without decreasing fasting blood insulin, mimicking the effects of calorie restriction [115]. In humans, a recent study reported an improvement of insulin sensitivity in plasma glucose in subjects with impaired glucose tolerance, after four weeks of daily resveratrol administration (1 and 2 g/day), showing no differences between doses [116]. Regarding resveratrol's potential to mimic the beneficial effects of calorie restriction, in a recent human study on obese individuals who were administered 150 mg/day of resveratrol for 30 days, it induced similar metabolic changes as those achieved with CR, such as a reduction in sleeping and resting metabolic rate and an increase of AMPK, Sirt1, PGC-1*α*, and citrate synthase activity [117]. However, the above mentioned controversy still exists in humans [118].

Another effect attributed to resveratrol is the protection against the vulnerability associated with frailty [119]. It seems to act as a neuroprotective agent due to its anti-inflammatory activity and its ability to decrease the levels of tumor necrosis factor, cyclooxygenase 2, inducible nitric oxide synthase, and interleukins [120]. Such neuroprotective activity could also be mediated by increasing Sirt1 activity, because this enzyme is thought to increase neurotrophic factors, such as brain-derived neurotrophic factor (BDNF). Specifically, in mice with a phenotype of accelerated aging (SAMP8) an increase of Sir and BDNF is produced when rats are calorie-restricted [121].

## 5. Conclusions

According to the bibliography, we could state that resveratrol has many beneficial properties. Its small molecular structure and polyphenolic character endow resveratrol with antioxidant properties and the ability to bind to organic compounds present in many organisms, such as hormone receptors and enzymes. This ability to interact with biological molecules provides resveratrol with multiple biological activities that are evident and clear when studied* in vitro*. These include beneficial effects against tumor processes, cardiovascular parameters, and longevity. However, some discrepancies have been observed in* in vivo* studies.

The concentrations used* in vitro* are too high to be reached in the organism after red wine consumption. However, it is possible to achieve such high concentration of resveratrol in plasma by administering resveratrol supplements and that is how many of the* in vitro* results have been verified in animal tests. However, if nutrients containing resveratrol are used to test these effects* in vitro*, the results show little biological activity. This is due to the small amount of resveratrol present in natural products and its low bioavailability limits its activity in the target tissues. The issue of bioavailability is determined by its rapid elimination and the fact that its absorption is highly effective, but the first hepatic step leaves little free resveratrol. In fact, free resveratrol can even bind to plasma proteins that could serve as a reservoir.

Clarifying aspects like stability and pharmacokinetics of resveratrol metabolites would be fundamental to understand and apply the therapeutic properties of resveratrol.

Further research into resveratrol uptake, cellular destination, metabolism, and stability of the original molecule and that of its metabolites is needed to elucidate its biological activity and it would be crucial to take advantage of the efficiency of its properties [30].

## Figures and Tables

**Table 1a:** (a) Clinical trials (humans)

Article	Study type	Administered dose	Treatment time	Blood resveratrol	Dietary dose	Relevance
[37]	*In vivo*: human	Oral: 25 mg; intravenous: 1.5 mg	Once	<5 ng/mL	NO	Resveratrol is quickly metabolized

[49]	*In vivo*: human	Oral: 25 mg	Once	10 to 40 nmol/L	NO	*In vitro* anticancer and anti-inflammatory effects of the free polyphenols are irrelevant *in vivo *

[45]	*In vivo*: human	Diet: 300 mL (0.82 mg/L) 600 mL (3.2 mg/L) 600 mL (0.48 mg/L)	Once	Not detected	YES	The observed protective effect on cardiovascular diseases associated with a moderate consumption of wine may be due to the whole polyphenols contained in wine and not to resveratrol alone

[54]	*In vivo*: human	Oral: 2 times/day 2000 mg	16 days	1274 ± 790 ng/mL	NO	A high-fat meal decreases resveratrol absorption

[39]	*In vivo*: human	Oral: 1 g	Once		NO	A rapid, sensitive, and accurate method for the analysis of resveratrol and its metabolites in human plasma and urine

[50]	*In vivo*: human	Oral: 6 times/day 25 mg 50 mg 100 mg 150 mg	Two days	3.89 ng/mL 7.39 ng/mL 23.1 ng/mL 63.8 ng/mL	NO	Repeated administration was well-tolerated but produced relatively low plasma concentrations of *trans*-resveratrol, despite the different administrated doses; *trans*-resveratrol pharmacokinetics showed circadian variation; bioavailability was higher after morning administration

[55]	*In vivo*: human	Wine in diet			YES	A moderate wine consumption (alcohol) may be one explanation for protection from coronary heart disease

[116]	*In vivo*: human	Oral: 1 g/day 1.5 g/day 2 g/day	28 days		NO	Resveratrol improves insulin sensitivity in subjects with impaired glucose tolerance

[117]	*In vivo*: human	150 mg/day	30 days	182.59 ± 30.33 ng/mL	NO	Resveratrol supplementation induces metabolic changes in obese humans, mimicking the effects of calorie restriction

**Table 1b:** (b) *In vivo* (animals)

Article	Study type	Administered dose	Treatment time	Relevance
[51]	*In vivo*: F344 rat	Oral: 200 *μ*g/kg/day	100 days	A protective role of resveratrol in colon carcinogenesis.

[52]	*In vivo*: F344 rat	Orally or intraperitoneally: 1 mg/kg 2 mg/kg	16 weeks 20 weeks	Resveratrol may be a promising natural anticarcinogenesis agent for the prevention and treatment of human esophageal cancer

[53]	*In vivo*: Sprague Dawley rat	Diet: 200 *μ*g/rat/day	120 days	Resveratrol suppresses 7,12-dimethylbenz(a)anthracene induced mammary carcinogenesis

[60]	*In vivo*: APfSD rat *In vitro*: Cos-1 cells hER-*α* Yeast	Oral or subcutaneous: 0.03–120 mg/kg/day		Weak estrogenicity of the red wine constituent resveratrol

[62]	*In vivo:* weanling rat	Oral: 1, 4, 10, 40, and 100 *μ*g/day	Six days	Resveratrol has little or no estrogen agonism on reproductive and nonreproductive estrogen target tissues and may be an estrogen antagonist

[103]	*In vivo*: streptozotocin-induced diabetes mellitus Sprague-Dawley rats	Oral: 0.75 mg/kg three times a day	Eight weeks	Resveratrol improves energy metabolism and reduces protein wasting

[99]	*In vivo*: rabbit	Oral: 4 mg/kg/day	12 weeks	Resveratrol inhibits platelet aggregation

[115]	*In vivo*: *Microcebus murinus *	Diet: 200 mg/kg/day	21 months 33 months	Resveratrol affects insulin sensitivity by improving glucose tolerance

[105]	*In vivo*: *Caenorhabditis elegans* *Drosophila melanogaster *	Diet: 100 *μ*M	Whole life	Resveratrol activates sirtuins in *Caenorhabditis elegans* and *Drosophila melanogaster* and extends their lifespan

[108]	*In vivo*: *Drosophila melanogaster *	Diet: 50–500 *μ*M	Whole life	Resveratrol extends lifespan

[109]	*In vivo*: *Caenorhabditis elegans *	Diet: 100–1000 *μ*M	Whole life	Lifespan extension in *C. elegans* is mediated by sir-2.1

[82]	*In vivo*: *Apis mellifera *	Diet: 30–130 *μ*M	Whole life	Resveratrol significantly affects gustatory responsiveness and prolongs lifespan under normal oxygen conditions

**Table 1c:** (c) *In vitro *

Article	Study type	Administered dose	Relevance
[63]	*In vitro*: MCF-7 cells T47D cells MDA-MB-231 cells	3–10 *μ*M	Resveratrol exhibits variable degrees of estrogen receptor agonism in different test systems

[89]	*In vitro*: DU-145, PC-3, and JCA-1 human prostate cancer cells	25 *μ*M	Resveratrol negatively modulates prostate cancer cell growth

[90]	*In vitro*: HL-60 cells Hepa LcLc7 cells	11, 18, 21, 27 *μ*M	Resveratrol is a potential cancer chemopreventive agent

[91]	*In vitro*: MCF7 cells	10 *μ*M	Resveratrol blocks the aryl hydrocarbon receptor and has beneficial effects against some types of tumors

[92]	*In vitro*: MCF7 cells	10, 50, 100, 150 *μ*M	The anticancer effect of resveratrol is via BCL-2 and NF*κ*B

[93]	*In vitro*: human lymphoblast cells	2.5, 5, 10, 20, 40 *μ*M	The anticancer effect of resveratrol is via p53

[94]	*In vitro*: L1210-R2 murine lymphoblastic leukemia cells K-562 human myelogenous leukemia cells P-815 murine mastocytoma cells	0.1–1000 *μ*M	The anticancer effect of resveratrol is via inhibiting ribonuclease reductase

[95]	*In vitro*: murine 3T6 fibroblast	0.3–30 *μ*M	Reactive oxygen species and arachidonic acid might be involved in the control of 3T6 fibroblast growth by resveratrol

[98]	*In vitro*: human platelets	0.1, 1.0 and 10.0 *μ*M	*trans*-Resveratrol is an inhibitor of store-operated Ca^2+^ channels in human platelets. This accounts for the ability of *trans*-resveratrol to inhibit platelet aggregation induced by thrombin

[104]	*In vitro*: *Saccharomyces cerevisiae *	0–500 *μ*M	Resveratrol stimulates Sir2, thus increasing DNA stability and extending lifespan
